# The first cell-fate decision of mouse preimplantation embryo development: integrating cell position and polarity

**DOI:** 10.1098/rsob.170210

**Published:** 2017-11-22

**Authors:** Aleksandar I. Mihajlović, Alexander W. Bruce

**Affiliations:** Laboratory of Developmental Biology and Genetics (LDB&G), Department of Molecular Biology and Genetics, Faculty of Science, University of South Bohemia, Branišovská 31, 37005 České Budějovice, Czech Republic

**Keywords:** cell-fate, cell positioning and polarity, preimplantation mouse embryo

## Abstract

During the first cell-fate decision of mouse preimplantation embryo development, a population of outer-residing polar cells is segregated from a second population of inner apolar cells to form two distinct cell lineages: the trophectoderm and the inner cell mass (ICM), respectively. Historically, two models have been proposed to explain how the initial differences between these two cell populations originate and ultimately define them as the two stated early blastocyst stage cell lineages. The ‘positional’ model proposes that cells acquire distinct fates based on differences in their relative position within the developing embryo, while the ‘polarity’ model proposes that the differences driving the lineage segregation arise as a consequence of the differential inheritance of factors, which exhibit polarized subcellular localizations, upon asymmetric cell divisions. Although these two models have traditionally been considered separately, a growing body of evidence, collected over recent years, suggests the existence of a large degree of compatibility. Accordingly, the main aim of this review is to summarize the major historical and more contemporarily identified events that define the first cell-fate decision and to place them in the context of both the originally proposed positional and polarity models, thus highlighting their functional complementarity in describing distinct aspects of the developmental programme underpinning the first cell-fate decision in mouse embryogenesis.

## 1. An overview of preimplantation mouse embryo development

**Figure 1.**
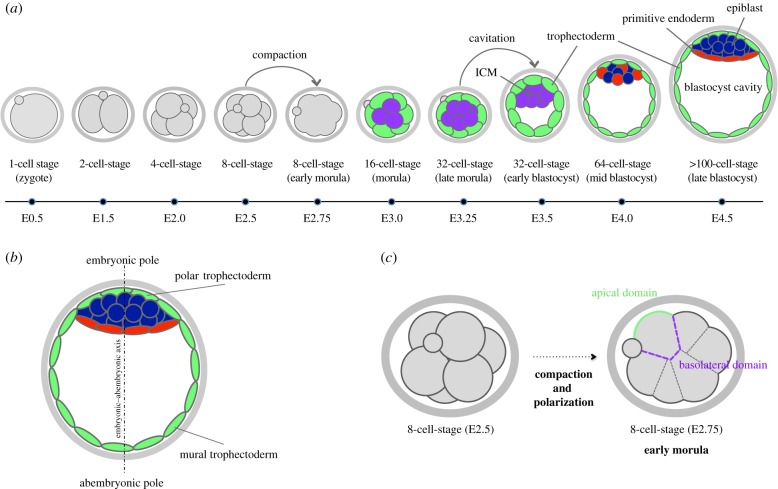
The preimplantation period of mouse embryo development. (*a*) The temporal sequence of events throughout preimplantation mouse embryo development with relevant embryonic stages and cell lineages generated as a result of the first and the second cell-fate decisions. (*b*) Orientation of the embryonic–abembryonic axis in the late blastocyst stage embryo (E4.5). Note the position of mural and polar trophectoderm at abembryonic and embryonic poles of the embryo, respectively. (*c*) A non-compacted 8-cell-stage embryo undergoing the first morphogenetic event (compaction) to develop into an early morula-stage embryo. Concomitantly, intracellular polarization is established as exemplified by the apical (green), and basolateral (purple) membrane domains of individual blastomeres.

Fertilization of the mouse egg, resulting in the formation of a zygote, marks the beginning of the preimplantation period of mouse embryo development. The zygote (1-cell stage) subsequently undergoes a series of asynchronous cell cleavage divisions, within its protective proteinaceous shell (the zona pellucida), without altering its overall cytoplasmic volume (figure 1*a*). As a consequence, preimplantation development proceeds through a number of intermediary stages defined by an increasing number of progressively smaller cells, known as blastomeres, and concludes with the derivation of the so-called blastocyst-stage embryo, capable of uterine implantation [1]. Such peri-implantation-stage blastocysts comprise an outer epithelium of extra-embryonic differentiating trophectoderm cells (precursors of the embryonic component of the placenta) encapsulating a fluid-filled cavity and an inner cell mass (ICM), itself comprising a second population of cavity-facing and differentiating epithelial cells, known as the primitive endoderm, and a deeper residing population of pluripotent epiblast cells, which serve as a source of progenitor cells for the development of the fetus proper (figure 1*a,b*).

Preimplantation mouse embryo development is driven by an as yet unidentified endogenous clock that ensures specific developmental events are associated with particular developmental cell cycles [2,3]. The first two cell divisions in mouse preimplantation development are significantly longer than those subsequent and last approximately 20 hours versus the 12 hours of later cleavage divisions [4]. Although the zygote initially relies upon stores of proteins and messenger RNAs (mRNAs), maternally provided to the oocyte, at the end of the 1-cell stage the zygotic genome becomes transcriptionally activated, initially by a restricted minor burst of activation that is then followed by a major burst at the end of the 2-cell stage, in a process known as zygotic genome activation (ZGA) [5]. Coincident with the onset of ZGA, the initiation of the destruction of maternal mRNAs begins. However, proteins that have been synthesized from maternal transcripts provided during oogenesis can persist, some until the end of the preimplantation developmental period [6].

Early cleavage-stage mouse embryos are highly adaptable and can withstand externally mediated perturbations such as the experimental removal, addition and rearrangement of blastomeres. For example, if one cell of a 2-cell-stage embryo is experimentally destroyed, the remaining blastomere is readily able to compensate for this loss and fully support subsequent development to term [3,7]. Additionally, the combination of two intact individual mouse preimplantation-stage embryos, or the aggregation of populations of previously dissociated/isolated blastomeres with themselves or with other embryos (not necessarily synchronized in terms of their developmental progression), can give rise to a single viable chimaeric adult mouse [8–10]. The plasticity with which mouse embryos are able to adapt to such experimental interventions is a consequence of the remarkably regulative nature of mouse embryo development; indeed this is one of the most distinguishing features of early mammalian development. However, it is important to note that as the embryo development progresses, such defining plasticity is gradually lost [8,11,12].

**Figure 2.**
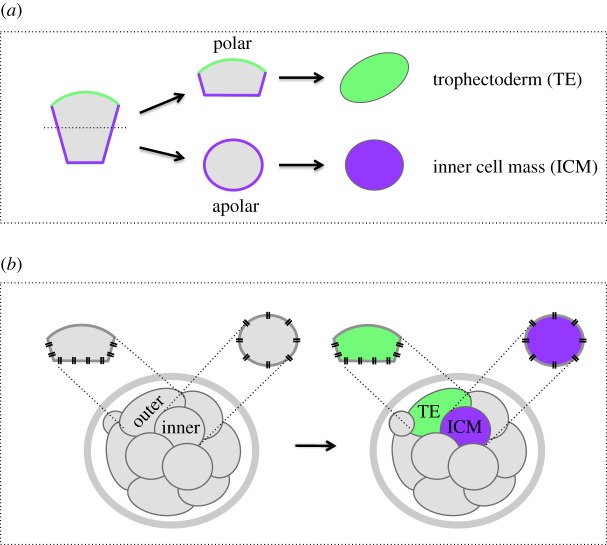
The classical ‘polarity’ and ‘positional’ models proposed to explain the first cell-fate decision. (*a*) A schematic representation of the ‘polarity’ model showing that the differences required to set the trophectoderm (TE) and the ICM cell lineages apart arise as a result of an asymmetric partitioning of polarized subcellular components between daughter cells (e.g. differential inheritance of apical and basolateral membrane domains) upon asymmetric cell division; solid green and purple lines, respectively, mark the apical and basolateral membrane domains, while the dashed black line marks the cell cleavage plane. (*b*) A schematic representation of the ‘positional’ model showing that the differences required for the segregation of the TE and the ICM cell lineages originate in the differential extent of cell-to-cell contact between individual blastomeres, corresponding to their relative position in the embryo; the sites of the cell-to-cell contact are highlighted with two parallel black lines, reminiscent of adherens junctions.

Although individual cells separated from the 4- or 8-cell-stage mouse embryo cannot independently develop beyond implantation [13,14], they are, nevertheless, able to contribute to all tissues when combined with other blastomeres in experimentally derived chimaeras, indicating that they still retain their full developmental potential [15–17]. This is not to state that such cells have not already begun the process of differing from each other (see below), but rather they have not yet reached a point at which their individual development potential has been irreversibly restricted. Indeed, up until the 8-cell stage, the blastomeres of early cleavage embryos are morphologically identical, each comprising both cell-contact-engaged basolateral membrane domains and contactless apical surfaces. However, such morphological identity does not necessarily translate into a paralogous homogeneity on the molecular or ultimately a cell-fate level. Indeed, a growing body of emerging evidence demonstrates the presence of distinct molecular differences between the constituent cells of pre-16-cell-stage embryos that have variously been reported to bias the ultimate cell fate of descendent progeny cells, with respect to populating the blastocyst ICM or the trophectoderm [17–23]. However, despite this potential for such early inter-blastomere heterogeneity to influence subsequent cell fate, it is clear that such cells are remarkably plastic and highly influenced by their cellular environment. Indeed, at the 8-cell stage, the extent of intercellular contact between neighbouring blastomeres increases while simultaneously the contact-free surface area decreases, as the embryo undergoes the first morphogenetic event in embryogenesis, known as compaction (figure 1*c*) [24]. As a result of compaction, adherens junctions form at the cell-to-cell contact sites, creating the embryonic structure commonly referred to as the morula, a term variously used to describe embryos at stages comprising the compacted late-8-cell stage to the developmental point prior to blastocyst formation at the 32-cell stage [25]. In parallel with compaction, blastomeres undergo a process of intracellular polarization, resulting in the asymmetric distribution of defined cellular components (discussed below), between the apical and basolateral membrane domains, thus defining the establishment of a radial, with respect to the embryo, apical–basolateral axis of cell polarity in each blastomere [26]. Importantly, it is at this stage that tight junction formation is also initiated, at the border between the apical and basolateral regions, that ultimately serves to delineate these two membrane domains [27], each of which are required to confer an appropriate cell-fate identity to subsequent cells. Indeed, the successful conclusion of both polarization and compaction is an essential prerequisite for the appropriate allocation of cell fate to the two morphologically and spatially distinct populations of blastomeres that arise as a consequence of the 8- to 16-cell and 16- to 32-cell-stage transitions, when cells become allocated to relatively inside/encapsulated or outside positions of the embryo [28,29]. This is because based on the initial differences in their position (outer/inner) and intracellular organization (polarized/non-polarized), such resultant cells will eventually segregate into one of two different cell lineages, trophectoderm or ICM, in a process typically referred to as the first cell-fate decision (and historically interpreted against the backdrop of two classical and overarching theories represented by the ‘positional’ and ‘polarity’ models—summarized in figure 2 and discussed in depth below). Accordingly, outer blastomeres that retain their intracellular polarity typically give rise to the trophectoderm, while non-polarized inner blastomeres, which lack components normally enriched at the apical domain, will become the ICM. However, it is important to note that such initial spatial segregation and differential polarization status is not immediately conveyed into the irreversible establishment of the trophectoderm and ICM lineages. This is perhaps best illustrated by the fact that blastomere aggregates made from populations of either exclusively inner or outer 16-cell-stage blastomeres are able to reconstitute the preimplantation developmental programme [30] and are capable of developing into normal and fertile mice, when transferred to the uteri of pseudo-pregnant female foster mice [12]. These data illustrate that both inner and outer 16-cell-stage blastomeres are still able to reprogramme their development in accordance to their relative position in the embryo and are thus able to give rise to both the trophectoderm and the ICM, demonstrating that they are not irreversibly committed to one or another cell lineage at this stage. Given the fact that 32-cell/blastocyst-stage blastomeres (assayed at the developmental point at which cavitation is first initiated) appear to lack similar plasticity in similar assays, it has been proposed that populations of outer and inner cells become irreversibly committed to the trophectoderm and the ICM by this stage, thus marking the point at which the first cell-fate decision can be argued to be finalized [12]. However, subsequent experiments have revealed that at least some blastomeres of such blastocysts retain their full potential [31] and, consistent with these data, it has recently been reported that the developmental potential of trophectoderm cells seems to be terminally restricted by the late 32-cell stage, whereas cells of the ICM only fully commit, becoming unable to give rise to the trophectoderm, during the 32- to 64-cell-stage transition [11].

At the 32-cell stage an osmotic gradient across the outer-residing and emerging trophectoderm lineage is created. This is caused by the combined influx of Na^+^ ions via apically localized Na^+^/H^+^ exchangers and efflux through basolaterally located Na^+^/K^+^ ATPases, which drives the transport of water across the trophectoderm, thus forming the fluid-filled cavity that is the defining morphological feature of the blastocyst [32–34]. Moreover, the gradual expansion of this cavity during blastocyst maturation is required to permit the embryo to hatch from within the zona pellucida and ultimately implant into the uterus. It is imperative that the maturation of tight junction formation between adjacent outer trophectoderm cells is concluded by this stage in order to form a functionally intact epithelium capable of maintaining the expanding blastocyst cavity [35,36]. The blastocyst cavity itself is asymmetrically positioned to one side of the embryo, thereby restricting the previously derived ICM population of cells to the opposite pole and as such defining the embryonic–abembryonic axis (figure 1*b*), whereby the position of the ICM delineates the embryonic pole. The part of the blastocyst trophectoderm that is in contact with the cavity (in the abembryonic region) is referred to as the mural trophectoderm, while the portion opposing it, covering the ICM, is known as the polar trophectoderm.

As referenced above and following the specification of the trophectoderm and the formation of the blastocyst cavity, two further ICM lineages, the pluripotent epiblast and the differentiating extra-embryonic primitive endoderm, are formed and spatially segregated from each other (figure 1*a,b*), as a consequence of the second cell-fate decision. The precursor cells of these two lineages are at first seemingly randomly distributed throughout the early blastocyst ICM, in what became known as the ‘salt-and-pepper’ pattern [37]. However, as the blastocyst develops, these cells are gradually segregated into spatially distinct epiblast and primitive endoderm compartments, via processes of cell sorting and programmed cell death [38,39], so that by embryonic day 4.5 (E4.5) of preimplantation development the primitive endoderm cells constitute a single-cell monolayer of intracellularly polarized cells in contact with the blastocyst cavity, while the epiblast remains as a mass of cells residing between the primitive endoderm and the overlying polar trophectoderm.

After completing a total of seven cleavage divisions, the mouse embryo emerges, from the zona pellucida, as a morphologically recognized and distinct structure called the late blastocyst, comprising three distinct cell lineages and capable of implanting into the uterus, thus marking the end of the preimplantation period of mouse embryo development. It is the aim of the first part of this review to focus upon and to summarize the relevant events, including recent mechanistic insights, that appear to underpin the first cell-fate decision of mouse preimplantation embryo development and then to place these in the wider context of the two key historically proposed theories, represented by the ‘positional’ [14] and ‘polarization’ [26] models (summarized in figure 2; discussed in detail below), originally put forward to describe this fundamental period in mouse development.

## 2. Compaction and polarization

Two hallmark events of 8-cell-stage embryo development that precede the formation of two spatially distinct cell populations and are critical for the first cell-fate decision are compaction and polarization. Although closely temporally linked, these two events, compaction, referring to intercellular organization of the embryo, and polarization, reflecting the intracellular organization of the individual blastomere, can be dissociated from one another.

### 2.1. Compaction

Compaction is the first morphogenetic event in mouse embryo development, during which initially spherical blastomeres change their morphology as they intensify intercellular contact and flatten against each other. As a result, previously observable intercellular boundaries become obscure as adherens junctions form at cell-to-cell contact sites.

Arguably, the most important protein involved in the process of compaction and the formation of adherens junctions is the cell adhesion molecule, epithelial cadherin (E-cadherin, hereafter referred to as E-cad; encoded by the *Cdh1* gene). Prior to compaction, E-cad is evenly present throughout the entire plasma membrane of all 8-cell-stage blastomeres. However, during compaction this subcellular localization changes as the adherens junctions begin to form, restricting E-cad protein to the basolateral cell-to-cell contact sites [40]. Indeed, the addition of specific antibodies that recognize E-cad antigens or the removal of Ca^2+^ ions from the embryo growth media (that prevents E-cad homophilic binding) ablates embryo compaction [40–42]. However, embryos in which the zygotic alleles of the *Cdh1* gene have been genetically removed are still able to compact normally, due to maternally provided stores of the protein, and only fail to form functional adherens junctions later, at the blastocyst stage [43,44]. The removal of the maternal *Cdh1* gene alone is sufficient to prevent cell adhesion but only delays the onset of compaction until the late morula stage [45]. It is only upon the removal of both maternal and zygotic *Cdh1* that embryos fail to compact at all [46].

Experimental evidence suggests that the protein components necessary for the initiation of compaction are already present in the blastomeres of 4-cell stage embryos, thus indicating that all the changes required for the onset of compaction are most probably regulated at the post-translational level [47,48]. Consistently, E-cad phosphorylation in preimplantation mouse embryos coincides with the onset of compaction [49], and precocious activation of Ca^2+^-phospholipid-dependent protein kinase C (PKC), upon treatment of 4-cell stage embryos with phorbol esters or synthetic analogues of diacylglycerides, induces premature compaction in a manner that is entirely dependent on E-cad [50]. Nevertheless, the inhibition of PKC activity itself, although associated with the aberrant localization of accumulated E-cad protein at the apical domain of the 8-cell-stage embryos, does not prevent embryo compaction *per se* [51].

Regarding the mechanical forces responsible for physically shaping the embryo, Fierro-Gonzalez and colleagues [52] have reported the existence of stage-specific and E-cad-dependent filopodia that they propose blastomeres employ in order to attach to the contactless apical domains of neighbouring cells. They postulate that this intensifies the required cell-to-cell contact and generates apical domain tension that maintains an elongated morphology in the filopodia-forming cell, thus controlling the cell shape changes required for appropriate embryo compaction, conclusions underpinned by both laser-induced ablation of E-cad-dependent filopodia and experimental downregulation of their integral protein components [52]. However, these conclusions have subsequently been challenged by Maitre *et al.* [53], who alternatively proposed that the initiation of embryo compaction is primarily driven by contractility of the actomyosin cortex that underlies the plasma membrane. Specifically, they assert that observable and pulsed contractions within the actomyosin cortex are responsible for generating an increase in the surface tension within contactless apical domains that is required for embryo compaction; moreover, this is an intrinsic/cell-autonomous property of the cell that is readily observable in isolated 8-cell-stage blastomeres and is independent of the presence of E-cad (as confirmed in blastomeres derived from combined maternal and zygotic *Cdh1* genetic knockout embryos [53]). Additionally, the authors also suggest that the role of E-cad during preimplantation mouse embryo compaction is actually to reduce the observed contractility and to direct it away from pre-existing sites of cell-to-cell contact. Notwithstanding such evidence, it is important to note that the role of filopodia and/or E-cad/cell adhesion in mediating compaction has not been undermined, rather that it is more likely they play a role in maintaining the compacted state of the embryo, as opposed to initiating compaction *per se*.

### 2.2. Polarization

Cell polarity is defined as a structurally and functionally asymmetric organization of cellular components that contributes to cell asymmetry and is preserved and transmitted through cell divisions (reviewed in [54]). This asymmetric organization implies an asymmetric distribution of protein factors between two distinct, apical and basolateral, plasma membrane domains and the formation of an apical–basolateral axis of cell polarity. Importantly, the asymmetric distribution of such factors permits their potential differential inheritance upon cell division and a consequent functional divergence between the two arising daughter cells; this divergence is eventually translated into different cell fates.

The establishment of cell polarity in the mouse preimplantation embryo occurs concomitantly with compaction and is initiated de novo at the 8-cell stage. The entire process, from the initial induction until the establishment of the matured and polar apical–basolateral axis, encompasses 3–5 hours of development [26]. Importantly, the establishment of cell polarity is independent of that of compaction, supported by the observation that blastomeres of combined maternal and zygotic *Cdh1* genetic knockout embryos (that fail to compact) are still able to polarize [46]. Moreover, as cell polarity is established in individual isolated blastomeres (deprived of cell-to-cell contact), the process can be considered as cell-autonomous [55,56]. However, it is important to state that this observation does not imply that during undisturbed development intercellular contacts do not play a role in the establishment of cell polarity. On the contrary, the importance of cell contact in the establishment of cell polarity has been appreciated for some time [26,27,57]. Namely, the apical pole has a tendency to form as far away as possible from the sites of cell-to-cell contact, suggesting that the orientation of the apical–basolateral axis is highly dependent upon the asymmetric intercellular contact patterns observable in the compacting 8-cell-stage mouse embryo. Indeed, a recently conducted study has corroborated these observations and has further demonstrated that the cell adhesion molecule E-cad is, surprisingly, dispensable for directing the orientation of the intracellular apical–basolateral axis [56]. Although polarization can be initiated in blastomeres that have been isolated from cell-to-cell contact or prevented from compacting, it is extremely difficult to experimentally induce polarity in blastomeres that are completely surrounded by other cells [26]. Therefore, by changing the cellular contact pattern of individual early 8-cell-stage blastomeres, it is experimentally possible to change the axis of polarity of each individual cell or to prevent them from polarizing at all [26].

One of the earliest documented events relevant for the onset of cell polarity establishment is the phosphorylation of the protein ezrin (Ezr), the role of which in the preimplantation embryo is to participate in the formation and stabilization of microvilli [58]. The individual blastomeres of non-compacted 8-cell-stage embryos are not polarized and are characterized by a round morphology and the presence of microvilli distributed evenly throughout their entire cell surface; however, as the embryo undergoes compaction and blastomeres polarize along their apical–basolateral axes, the microvilli become excluded from the cell-to-cell contact regions and eventually restricted to the contact-free apical membrane [59]. The subcellular localization of Ezr follows the dynamics of microvilli distribution and, prior to the onset of polarization and compaction, is likewise uniformly distributed throughout the cell membrane. However, upon its phosphorylation, Ezr becomes excluded from regions of cell-to-cell contact and accumulates at the apical membrane domain [60], thereby favouring microvilli formation only at the apical regions. The phosphorylation of Ezr at amino acid residue threonine-567 appears to be the main prerequisite for its polarized redistribution at the 8-cell stage, as substitution of this residue for alanine inhibits both the normal removal of Ezr from cell-to-cell contact regions and microvilli breakdown at the basolateral membranes. Importantly, it also prevents the appropriate formation of cell-to-cell contacts mediated by E-cad and associated embryo compaction [58]. There is some evidence to suggest that aPKC*ζ*/*λ* (atypical PKC, represented by the two isoforms zeta/*ζ* and iota/lambda/*ι*/*λ*, hereinafter commonly referred to as Prkcz/i or when necessary as Prkcz or Prkci individually) may be responsible for this phosphorylation event [51], albeit using an antiserum that not only recognizes phospho-Ezr but also phospho-forms of the related proteins radixin and moesin. Nevertheless, Prkcz/i-deficient embryos as well as embryos injected with a dominant-negative form of Prkci (that interferes with the function of both Prkcz and Prkci) appear to have no problem undergoing compaction [61]. Interestingly, it has been proposed that Prkcz/i itself might be regulated by the small GTPase RhoA (Ras homology family member A, guanosine triphosphate hydrolase) [51], the role of which in both compaction and polarization has been previously demonstrated via chemical inhibition [62].

Regardless of how it is initiated, experimental evidence suggests that maintenance of cell polarity involves the activity of polarity protein complexes. One of the earliest studies to investigate the presence of protein polarity factors demonstrates that components of the apical polarity protein/partitioning defective complex (aPKC–PAR complex), consisting of Pard3 (partitioning defective 3 homologue), Pard6 (partitioning defective 6 homologue) and Prkcz/i, as well as the basolateral domain marker of cell polarity Emk1 (ELKL motif kinase 1, also known as microtubule affinity-regulating kinase 2—Mark2; the mammalian homologue of PAR-1 in *Drosophila*), are all expressed in the preimplantation mouse embryo [63]. In addition, the presence of other components of the basolateral polarity complex, for example scribble (Scrib) and lethal giant larvae homologue 1 (Llgl1), has been reported [61,64–68]. Unfortunately, there is a relative paucity of knowledge regarding the transcriptional control of such polarity factor genes, although it has been reported that the expression of *Pard6b* is under the control of transcription factor AP-2 γ (Tfap2c) [69,70]; consistently, a great majority of the effects induced by *Tfap2c* downregulation, such as tight junction formation failure, polarity defects and an overall failure in trophectoderm cell lineage specification, are easily attributable to dysregulation of *Pard6b* expression [69–71].

The localization of all polarity proteins is highly dynamic and changes as development proceeds. Specifically, from the 2-cell stage until the early 8-cell stage, Pard6b and Emk1 are predominantly nuclear and only present at very low levels in the cytoplasm [63], while the localization of Prkcz/i at the 4-cell stage has been described as cytoplasmic [72]. However, after polarization Pard6b and Prkcz/i proteins become accumulated at the apical membrane, whereas Emk1 is conversely localized to the basolateral part of the cell membrane, alongside members of the scribble polarity complex, including Scrib itself and Llgl1 [61,63–65,68,72]. This mutually exclusive localization pattern of apical and basolateral polarity factors is highly dependent on the activity of downstream effectors of the small GTPase RhoA, known as the Rho-associated protein kinases (Rock1/2; represented by two isoforms Rock1 and Rock2). Indeed, chemical inhibition of Rock1/2 activity is associated with the mis-localization of both apical and basolateral polarity factors and impaired blastocyst formation. This is evidenced by ordinarily apically accumulated Pard6b and Prkcz/i proteins becoming atypically and uniformly distributed throughout the entire cell membrane and Scrib and Llgl1 basolateral markers being ectopically present at the apical membrane, functionally placing Rock1/2 activity upstream of polarization [65,66,73].

Additionally, the apical polarity proteins themselves have been shown to be directly involved in the process of tight junction formation [71,74] and blastocyst cavitation. Specifically, Pard6b and Prkcz/i proteins are known to be targeted to establishing tight junction regions, at the 8-cell stage, and are required for appropriate tight junction formation, as evidenced by the observed distribution of Tjp1 (tight junction protein 1) becoming severely abrogated after their experimentally induced downregulation [71,74]. Pard3 is also targeted to tight junctions, but only around the time when the blastocyst cavity is being formed, indicating that Pard3 may be involved in the maturation or maintenance of tight junctions rather than their establishment [63]. It is also possible that the observed eventual co-localization of Pard6b, Prkcz/i and Pard3 at junctional complexes is indicative of the formation of a functional aPKC–PAR complex, as observed in other systems exhibiting cellular polarity [63].

Taken together, the establishment of cell polarity is a prerequisite for the appropriate formation of tight junctions in preimplantation mouse embryo development. By contrast, the experimental disruption of cell polarity, either after *Pard6b* downregulation or Rock1/2 inhibition, has no impact on embryo compaction nor on the formation of adherens junctions [66,71].

## 3. The establishment of two spatially distinct cell populations

**Figure 3.**
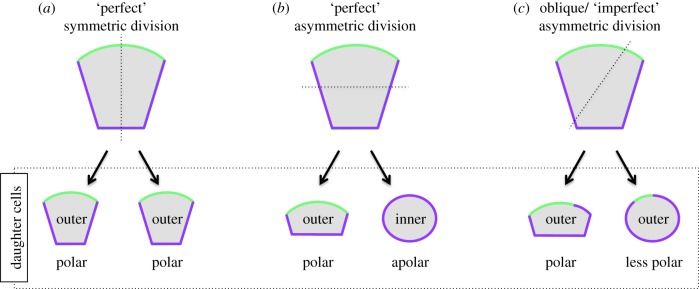
The possible outcomes for intracellular polarity and position following ‘perfect’ symmetric, ‘perfect’ asymmetric and oblique/‘imperfect’ asymmetric cell cleavage divisions. (*a*) A ‘perfect’ symmetric cell division (with the cleavage plane parallel with the apical–basolateral axis of polarity) produces two identical polar outer-residing daughter cells. (*b*) A ‘perfect’ asymmetric cell division (with the cleavage plane orthogonal to the apical–basolateral axis of polarity) produces a polar outer-residing and an apolar inner daughter cell. (*c*) An ‘imperfect’ asymmetric cell division (with the cleavage plane oblique to the apical–basolateral axis of polarity, at an angle that implies partitioning of the apical domain) produces two daughter cells that each inherit a certain but uneven portion of the apical domain in addition to an outside position immediately upon cell division. Note that in (*a*–*c*) solid green and purple lines, respectively, mark the apical and basolateral membrane domains, while dashed black lines mark cell cleavage planes.

As stated above, two spatially distinct populations of inner and outer cells are formed in the preimplantation mouse embryo, from the onset of 8- to 16-cell transition until the acquisition of the 32-cell stage (although highly infrequent cell internalizations have also been observed following transit of embryos to the 64-cell stage [75], but will not herein be considered further). The relevance of this spatial segregation is reflected in the fact that the two cell populations eventually acquire different fates, with the outer-residing cells becoming the trophectoderm while the inner cells form the nascent ICM. The exact mechanisms by which blastomeres acquire their particular relative position within the embryo remain an important and not fully resolved question. However, the initial acquisition of cell position within the embryo can be considered the direct consequence of division orientation (figure 3). For example, in cases where the mitotic spindles of 8-cell or outer-residing 16-cell-stage blastomeres are oriented perpendicular to the apical–basolateral axis, the resulting cleavage plane is parallel to it and generates two daughter cells that each inherits both apically and basolaterally distributed components. Such ‘conservative’ or ‘symmetric’ cell division results in the generation of two seemingly identical daughter cells that will in most cases retain polarized intracellular organization and occupy the outer positions of the embryo (figure 3*a*). However, if the mitotic spindle becomes oriented parallel to the apical–basolateral axis, the resulting cleavage plane is perpendicular to the same axis and results in the generation of two different daughter cells (figure 3*b*). As such, one cell retains the apical surface of the parental cell, remains polarized and is positioned on the outside of the embryo. Conversely, the other cell inherits the basolateral region of the parental cell, becomes apolar and is positioned in the inside compartment of the embryo. This type of division, in which two different daughter cells are generated, is called a ‘differentiative’ or ‘asymmetric’ division (extensively reviewed in [76]). Given that cell division orientation has a direct impact on the number of inner and outer cells generated in the embryo (and consequently the ratio of trophectoderm and ICM cells), the question that follows is whether division orientation is in anyway regulated or is it a completely random/stochastic process.

It has previously been hypothesized that cell shape (influenced by intercellular contact) and/or intrinsic organization of the cell (the extent of cell polarity/size of the apical domain, nuclear position or cytoskeleton) might influence division orientation [29]. However, the fact that there exists a highly variable ratio between the number of inner and outer cells, observed among individual embryos at the 16-cell stage, has led to the proposal that division orientation during the 8- to 16-cell transition is not under any significant regulation [77]. Interestingly, a more recent study suggests that nuclear positioning is correlative with predicting the orientation of an ensuing cell division, specifically reporting how at the early 8-cell stage most cell nuclei are located in the proximity of the apical domain, but as development progresses a certain portion of the nuclei become repositioned to the basolateral domain. Such differential localization was reported to be associated with a trend by which blastomeres with nuclei residing close to the apical surface are statistically more likely to undergo conservative/symmetric cell divisions (rather than differentiative/asymmetric ones), while their counterparts, in which nuclei had been repositioned to a more basolateral region, did not exhibit any bias in the orientation of their division [78]. Thus, this observation suggests the possibility that the orientation of cell division at the 8- to 16-cell stage may not be a completely randomized process. Indeed, in support of this view, a recently published study of Korotkevich and colleagues [56] reported the existence of a positive correlation between the size of the apical domain and the angle of the mitotic spindle in 8-cell-stage embryo blastomeres. Accordingly, blastomeres with apical domains that are concentrated in a smaller area exhibit mitotic spindles that are more precisely aligned with the radial axis of the embryo, thus suggesting that the division orientation might be under the control of apically localized polarity factors at this stage [56]. In addition, it has recently been proposed that the extent of cell-to-cell contact could also affect cell division orientation [79].

Interestingly, embryos that generate a relatively high number of inner cells by the 16-cell stage produce a proportionally lower number of newly internalized inner cells by the 32-cell stage and vice versa, thus ensuring the generation of a relatively constant number of inner and outer cells, between individual embryos, by the 32-cell/early blastocyst stage [29]. It is tempting to speculate that one potential mechanism of ensuring such a constant ratio of inner and outer cells might arise as a result of cell-shape-mediated control of cell division orientation, at the 16- to 32-cell-stage transition. Namely, in 16-cell-stage embryos that comprise a relatively large number of inner cells, the outer population would exhibit a more stretched morphology in order to encapsulate the inner cell population. This would result in the shortest possible plane of cell division aligning with the radial axis of the embryo. In contrast, the equivalent outer cells in embryos with relatively fewer inner cells would be more compressed and more likely to have a short cell division plane perpendicular to the radial axis. Given that it has been shown that frog embryo blastomeres preferentially divide across their shortest cell axis [80], it is possible a similar mechanism in 16-cell-stage mouse outer blastomeres may exist. Specifically, the outer cells of 16-cell-stage embryos with relatively fewer numbers of inner cells would preferentially divide in a manner to generate the additional inner cells required to regulate ICM cell number by the early blastocyst stage, and vice versa.

Although historically it has been considered that individual blastomeres acquire their position mainly as a direct consequence of division orientation, several recent studies suggest this may not entirely be the case. Namely, blastomeres do not necessarily maintain the initial/original position they acquired immediately upon cell division but instead actively position/sort themselves within the embryo as development proceeds [81–83]. Interestingly, although it has been reported that roughly three-quarters of blastomeres undergo an asymmetric division during the 8- to 16-cell-stage transition [56,81], it appears that only a fraction of inside cells are produced directly as a result of what may be considered a ‘perfect’ asymmetric cell division (i.e. one in which the inner cell is immediately allocated to an entirely encapsulated inner position that it then maintains). Instead, a significant proportion of inner cells seems to be generated by a process of internalization (sometimes unhelpfully referred to as engulfment; the process by which one cell internalizes another cell within itself) of apolar cells that initially occupied an outer position within the embryo as a whole [81,82]. Such cells appear to arise due to the fact that in reality the orientation of the cleavage plane is typically oblique rather than exactly perpendicular or parallel to the apical–basolateral axis of polarity (figure 3*c*). Such oblique divisions allow the apical domain to be unevenly partitioned between daughter cells (in a process that in a sense could be classified as being ‘imperfectly’ asymmetric) while simultaneously permitting both cells to achieve/acquire an outer position in the embryo, immediately consequent to the completion of cytokinesis [81]. As inferred above, such asymmetric partitioning of the apical domain is able to generate inter-blastomere heterogeneity among outer cells in the relative ‘extent’ of their individual apical–basolateral polarity (whether this is directly inherited *in situ* or re-established after the division is not currently clear), which is subsequently responsible for the internalization of less polarized outer cells [53], although it is theoretically possible that heterogeneity in terms of their adhesive properties may also be a contributing factor. Therefore, it is perhaps a moot point as to how cell division orientations in the preimplantation mouse embryos should be classified. One possible suggestion may be to classify such divisions on the basis of the extent to which a daughter cell inherits apical domain components. As an alternative, divisions that never result in a daughter cell being internalized could be considered as truly ‘symmetric’, whereas all others, by default, would be ‘asymmetric’ due to the fact that one daughter cell will eventually become internalized, despite initially occupying an outer position in the embryo.

The question that follows is why would less polar/apolar cells be disadvantaged over polar cells in retaining an outer position within the embryo? The first clue came from the observation that, prior to internalization, apolar cells are characterized by an increased cortical tension attributed to an increased level of detectable phospho-myosin light chain II (pMlc2/Myl9) [81]. Indeed, two subsequent and independent studies have since confirmed that the driving force for cell internalization does originate from differences in actomyosin contractility between neighbouring blastomeres, in addition to highlighting the role of Myh9 (myosin heavy chain 9, also known as non-muscle myosin heavy chain IIa) in this process [53,82]. Namely, the accumulation of phosphorylated myosin in the proximity of the contactless domain of apolar outer cells is responsible for inducing apical constriction required to internalize the cell [82]. Moreover, it appears that, in this context, the role of the apical polarity proteins is to oppose such actomyosin-induced contractility at this subcellular location, thus preventing internalization [53]. This conclusion is supported by the observation that phosphorylated myosin, ordinarily found restricted in the proximity of the presumptive tight junction region in polar outer 16-cell-stage blastomeres (A.I. Mihajlovic and A.W. Bruce, unpublished observations), accumulates and spreads across the entire apical domain in Prkcz/i-deficient blastomeres [53,74]. Thus, owing to the heterogeneity between blastomeres in the relative size of the generated apical domains by the onset of the 16-cell stage, less polar cells are characterized by increased contractility in comparison to their neighbouring polar cells and, as a result, they are more susceptible to internalization [53,82].

Importantly, these recent findings are able to reconcile two pre-existing and seemingly opposing observations, i.e. that while the experimental disruption of Prkcz/i in isolated 16-cell-stage blastomere doublets (2/16-cell doublets) after overexpression of a dominant negative form of Prkci forces blastomeres to exclusively undergo symmetric divisions [64] and global disruption of Prkcz/i across all cells of the developing preimplantation embryo, either using microinjected siRNA constructs (targeting Prkci transcripts) or a dominant-negative form of Prkci, results in an increased number of outer cells in 16-cell-stage embryos [74], the clonal dysregulation of Prkcz/i activity (also using the dominant-negative construct approach) biases the dysregulated cell clone into preferentially taking the inside position within the embryo [84]. In the light of the stated recent reports relating to polarity and cell internalization, this most probably reflects the fact that upon clonal dysregulation of Prkcz/i activity/function, the affected cell clones exhibit relatively increased contractility in their apical domains, when compared to their non-dysregulated neighbours, and are thus forced to internalize, whereas upon global dysregulation of Prkcz/i activity/function in 16-cell-stage embryos no such heterogenic contractile driving force exists. In addition, the experiments performed on isolated 2/16-cell doublets in which Prkcz/i activity/function has previously been dysregulated (using the dominant-negative form of Prkci) also demonstrated that the presence of a polarized cell is required in order to surround the apolar one [64]. Thus, a complete absence of polarized neighbouring cells in embryos with globally dysregulated Prkcz/i might provide an alternative/additional explanation for the observed phenotype. Taken together, it is tempting to speculate that the preferential internalization of such experimentally dysregulated Prkcz/i cell clones is representative of a more extreme manifestation of developmental processes ordinarily operative in unperturbed embryos, specifically that naturally occurring heterogeneity in the extent of apical polarity between individual blastomeres, generated by oblique division orientations, informs a cell's probability to be internalized or remain in an outer position. Notwithstanding these observations, it is intriguing that despite the reported deficit of inner cells in intact 16-cell-stage embryos after global dysregulation of Prkcz/i, an assay of similarly treated embryos at the 32-cell stage reveals no difference in the number of inner cells [74], suggesting that polarity or at least Prkci-independent mechanisms ensuring the required number of inner cells are operative by this time.

In summary, the relative position of a blastomere within a 16-cell-stage embryo is initially determined by division orientation of the parental cell that generated it and is potentially under the control of the factors localized to the apical domain. In addition, as a result of oblique cleavage division planes (with respect to the axis of intracellular apical–basolateral polarity), the asymmetric partitioning of the apical domain produces outer-residing 16-cell-stage blastomeres with differently sized apical domains and, consequently, differing extents of actomyosin contractility. These differences in contractility trigger cell sorting, so that less polar cells end up taking the inner positions of the embryo. Thus, the ultimate position of a blastomere in the preimplantation mouse embryo is highly dependent upon the extent of its own intracellular levels of apical–basolateral polarization, as determined by the inheritance of apically localized factors.

## 4. The interpretation of positional and polarity cues via the Hippo-signalling pathway

The appropriate interpretation of positional cues (provided by cell-to-cell contact) and the extent of cellular polarity, which together underpin the appropriate execution of specific cell-fate programmes, has been shown, at the molecular level, to be highly dependent upon the differential activation of the Hippo-signalling pathway. Indeed, it was Nishioka *et al.* [85] who first discovered the involvement of the Hippo-signalling pathway, by demonstrating its activity within inner cells and suppression in outer cells of the embryo, from the 16-cell-stage onwards. Specifically, the authors showed that it is the selective activation of the Hippo-pathway component serine/threonine protein kinases Lats1/2 (large tumour suppressor kinase 1 and 2), within completely surrounded inner cells that do not possess a cell contactless domain, that leads to the phosphorylation-dependent cytoplasmic sequestration of the transcriptional co-activator protein Yap1 (a transcriptional co-activator of Tead4/TEA domain transcription factor 4—itself required to activate transcription of trophectoderm-related genes [86,87]), whereas in outer cells a failure to activate Lats1/2 kinases permits unphosphorylated Yap1 to enter the nucleus and enables Tead4-dependent transcription of trophectoderm-required genes (although apolar outer cells, or those with comparatively small contactless apical domains, are able to activate Lats1/2). Thus, such spatially distinct and differential activation of the Hippo-signalling pathway ensures that only outer cells can express trophectoderm genes, while inner cells are prevented from such inappropriately activated gene transcription, given that they will not contribute cells to any future trophectoderm [29].

Since this landmark work [85], several other studies have demonstrated the involvement and fundamental importance of other Hippo-signalling pathway components during the first cell-fate decision [61,88–90]. These include two independent studies that highlight the central importance of the angiomotin (Amot) protein and the regulation of its subcellular localization in relation to differential Hippo-signalling pathway activation between inner and outer cells [61,89]. Amot is an activator of the Lats1/2 kinases and is first expressed as a weakly detectable immuno-fluorescence signal on the apical plasma membrane of 8-cell-stage embryos. However, from the 16-cell stage, Amot protein localization in newly derived inner cells is distinct from that of outer cells, being present throughout the entire plasma membrane, while in outer cells it is expressed at a relatively lower level and remains restricted to the apical domain [61,89]. Crucially, it is this differential pattern of Amot protein distribution between spatially distinct inner/apolar and outer/polarized cells that is of paramount importance in establishing the differential Hippo-pathway activation responsible for driving the first cell-fate decision. Specifically, in order to activate the Hippo-signalling pathway, Amot is required to associate with Lats1/2 kinases located at adherens junctions that, as stated above, form at basolateral regions of cell-to-cell contact after embryo compaction. However, in outer cells Amot is sequestered to the polarized apical domain and is thus prevented from associating with Lats1/2 [61]. As a consequence, Hippo-signalling is inhibited in outer cells and results in low levels of Lats1/2 activity that in turn is insufficient to drive phosphorylation-dependent Yap1 cytoplasmic retention. Thus nuclear-accumulated Yap1 is free to associate with Tead4 and to drive appropriate transcriptional activation of trophectoderm lineage-specific genes and transcription factors, such as *Cdx2*, to first specify and then reinforce trophectoderm cell fate in outer cells [85]. Conversely, in the apolar inner cell population, Amot is not able to be sequestered away from adherens junctions, and thus Lats1/2 and the Hippo-signalling pathway are activated and transcriptional activation of trophectoderm-specific genes is prevented [85].

The appropriate establishment of apical–basolateral cell polarity in outer cells is an absolute prerequisite for correct localization of Amot and its sequestration to the apical domain. Indeed, direct experimental disruption of either apical or basolateral polarity proteins results in the ectopic mis-localization of Amot to adherens junctions and the aberrant activation of the Hippo-signalling pathway, as evidenced by cytoplasmically localized and phosphorylated Yap1 [61]. Moreover, such a phenotype can be replicated by the pharmacological inhibition of the RhoA effector kinases, Rock1/2 [65,66], presumably as these phenotypes are also associated with a breakdown in outer cell apical–basolateral polarity. Interestingly, the fact that Yap1 is also phosphorylated, as a result of Hippo-signalling pathway activation, in naturally occurring apolar outer cells (in spite of the presence of contactless domain [64]) highlights the importance of a polarized cell organization and the presence of a functional apical domain in the ordinary negative regulation of the Hippo-signalling pathway in outer cells [81], indicating that outer cell apical Amot localization is not merely the sole function of possessing a contactless domain. It is noteworthy that although disruption of cell polarity at the 16-cell stage induces ectopic Hippo-signalling pathway activation in outer cells, the effects on Yap1 localization and *Cdx2* expression are relatively weak, when compared with equivalently disrupted embryos assayed at the 32-cell stage [64,66]. In line with these observations, the existence of potentially additional molecular mechanisms of Hippo-pathway activity/Yap1 subcellular localization regulation that work in parallel with the well-described apical cell polarity mechanism at the 16-cell stage has been proposed [64], although no evidence is yet forthcoming.

As stated above, the absence of a contact-free apical domain in derived inner cells permits the association of Amot with components of the adherens junctions complex and results in Lats1/2, and hence Hippo-signalling pathway, activation [61]. An elegant study has demonstrated the mechanistic requirement of the protein Nf2/Merlin (neurofibromin 2/moesin–ezrin–radixin-like protein) during this activation process. Specifically, in the absence of Nf2, embryos are unable to activate Hippo-signalling and hence ectopically localize Yap1 to the nuclei of ICM cells [88]. Thus, it has been proposed that Nf2, which itself is capable of binding to the adherens junction component Ctnna1 (α-catenin [91]), is required to mediate the interaction between Amot and the adherens junction complex [88]. Indeed it has been shown that the initial interaction of Amot with the adherens junction complex is further stabilized by a Lats1/2-dependent phosphorylation of Amot, which reduces its affinity for cortical filamentous actin (F-actin), thus promoting robust levels of Hippo-signalling activation [61]. The activation of Lats1/2 is subsequently responsible for the phosphorylation of Yap1 (at serine 112), which in turn promotes its cytoplasmic retention, via a phospho-dependent interaction with members of the cytoplasmic scaffold protein 14-3-3 family. Consequently, it is the retention of Yap1 in the cytoplasm that prevents the formation of any active transcriptional complexes with nuclear Tead4 and thus inappropriate expression of trophectoderm lineage-specific transcription factors [85]. However, it is important to note that such inhibition of trophectoderm-specific gene transcription is not the only known role for the activation of the Hippo-signalling pathway in inner cells. This is exemplified by the fact that activated Hippo-signalling is required to drive the expression of the pluripotency-related transcription factor gene *Sox2* [92], indicating a dual role for activated Hippo-signalling in derived inner cells, specifically suppressing inappropriate trophectoderm-specific gene expression and promoting the pluripotent state of the ICM, from which the epiblast is ultimately derived. In line with this observation, single-cell mRNA and protein expression analyses have revealed that *Sox2* expression is restricted to the inner cell population at the 16-cell stage, thus making Sox2 the earliest reported marker of the ICM [92,93]. Moreover, as inner cells can be derived following the embryo's transition from either the 8- to 16-cell or 16- to 32-cell stages (each separated by approximately 12 hours of developmental time), it raises the possibility that those inner cells which are derived comparatively earlier are more primed to ultimately yield the pluripotent epiblast cell lineage than their counterparts derived comparatively later, by virtue of a developmentally earlier and Hippo-pathway-dependent activation of *Sox2* expression. Indeed, it is equally possible that the relatively later derived inner cells are compromised to contribute to the epiblast as a consequence of being derived from outer 16-cell-stage blastomeres in which Hippo-signalling was repressed (therefore antagonizing *Sox2* expression) and differentiation towards the trophectoderm lineage was promoted. Such cells could be potentiated, once internalized some 12 hours later than the initial population of inner cells, to populate the differentiating primitive endoderm lineage. Thus, in such a manner the refinements in the spatiotemporal activation/suppression of the Hippo-signalling pathway (under the control of apical–basolateral polarization) may underpin the derivation of all three blastocyst cell lineages: trophectoderm, primitive endoderm and epiblast. Although slightly outside the scope of the current review and its focus on what we classically term the first cell-fate decision, it is interesting to note the presence of published data favouring such a Hippo-dependent mechanism of deriving the three blastocyst lineages [67,75,92–95].

In summary, the described published data serve to illustrate that individual blastomeres of the developing preimplantation mouse embryo are able to appropriately interpret their relative spatial/positional information, with respect to the inside–outside/radial axis of the embryo, and via a process of differentially regulated Hippo-signalling pathway activation. Moreover, this process is under the mechanistic control of the relative extent of intracellular apical–basolateral polarization (inter-blastomere heterogeneities which are influenced by the orientation of preceding cell cleavage divisions), thus permitting the germane regulation of cell lineage-specific gene expression, required to derive the first distinct cell lineages of mouse embryo development.

## 5. Re-evaluating both the ‘positional’ and ’polarity’ models of the first cell-fate decision and their relationship to each other

Historically, two models have been proposed to explain how the first cell-fate decision of preimplantation mouse embryogenesis is taken. The ‘polarity’ model (figure 2*a*) proposes that upon an asymmetric division of a polarized cell (be it at the 8-cell stage or an outer-residing blastomere at the 16-cell stage), the resulting daughter cells inherit differing amounts of cell-fate determinants that will later decide their ultimate fate [26,55]. The second model, termed the ‘positional’ or ‘inside–outside’ model (figure 2*b*), proposes that, based on their position (i.e. encapsulated inside or on the outside of the embryo), blastomeres are exposed to different micro-environments (perhaps reflected in differential cell contacts) that later become translated into different cell fates [14].

The establishment of cell polarity is a prerequisite for the initial divergence between the two early blastocyst cell lineages. In accordance with the polarity model, daughter cells that inherit a functional apical domain replete with the associated enriched apical factors (that, without documented exception, reside exclusively on the outer surface of the embryo) will remain polarized and are destined to become the trophectoderm, while the apolar daughter cells yield the ICM. In agreement with this model, a recent study has confirmed that the inheritance of an apical domain alone is sufficient to instruct a blastomere to acquire a trophectoderm cell fate [56]. This was elegantly demonstrated by the technically demanding transplantation of apical factor-enriched apical domains between isolated populations of polarized and apolar 8/16-cell-stage blastomeres, thus resulting in apical restriction of Amot protein, with consequent nuclear accumulation of Yap1 capable of instructing the previously apolar and apical domain-receiving cell to initiate a trophectoderm-specific transcriptional programme [56]. However, as valid as the polarization model appears, it can be argued that it is incomplete. This is because it suggests that the apolar sister blastomere that results from an asymmetric division is, by default, destined to become a cell of the putative ICM, which does not necessarily have to always be the case. To clarify, if a cell immediately acquires an inside position as a direct consequence of the completed cytokinesis in which it was generated, it will indeed stably contribute to the forming ICM. However, an apolar cell that initially acquires an outer position, following completion of the cell division, can either become internalized to populate the emerging ICM [81,82] or can repolarize, as has been reported using a fluorescently tagged Ezr construct as a reporter of apical polarity [56], and thus contribute to the trophectoderm cell lineage (although the actual frequency at which such repolarization events occur is a matter of contention [81]). Therefore, the fate of the apolar cell can be thought of as conditional and highly depends on its ultimate position in the embryo (what mechanistically triggers such outer-residing cells to initiate their repolarization is not clear, although as there is scant documentation that any inner cell would localize apical polarity factors to their plasma membrane, this suggests that the deciding factor must be the presence of a contactless domain, i.e. a reflection of position, potentially analogous to the de novo polarization of late 8-cell-stage blastomeres).

Importantly, the positional model alone also does not explain the segregation of the two early blastocyst cell lineages in their entirety. In agreement with the positional model, outer-residing cells are destined to become the trophectoderm, while inner cells become the ICM [14]. Indeed, the inner position in the embryo can be considered to impose a certain restriction with respect to a blastomere's intracellular organization that ensures inner cells will ultimately become the ICM, specifically that inner cells must be apolar by default (at any time), as it is impossible to induce polarity in completely surrounded cells (as discussed above). However, upon isolation, inner apolar cells are able to repolarize [46,96]. These data strongly imply that one relevant role of cell internalization *per se* is to prevent the inappropriate induction of a polarized cell organization, thus simultaneously ensuring, at the molecular level, that intracellular Amot protein is able to associate with the necessary molecular components (as described above at adherens junctions) to activate the Hippo-signalling pathway (due to the absence of an apical domain and its associated ability to sequester Amot) and allow the acquisition of an ICM cell fate. In addition, it is imperative that by the 32-cell stage, only polarized cells occupy an outer position in the embryo to allow the formation of a functional epithelium and the expansion of the blastocyst cavity. Nevertheless, as described, the presence of apolar outer cells is temporarily tolerated during the 16-cell stage. These cells that, according to the strictest interpretation of the positional model, are destined to become the trophectoderm are able to internalize and thus become the ICM. This implies that the positional model is also insufficient to fully explain the segregation of the early blastocyst lineages, in relation to such cells. Therefore, while the positional model holds true for all inner and polar outer cells, it also fails to predict the fate of outer-residing apolar cells. As a consequence, both models alone are insufficient to entirely explain how the first cell-fate decision is made.

**Figure 4.**
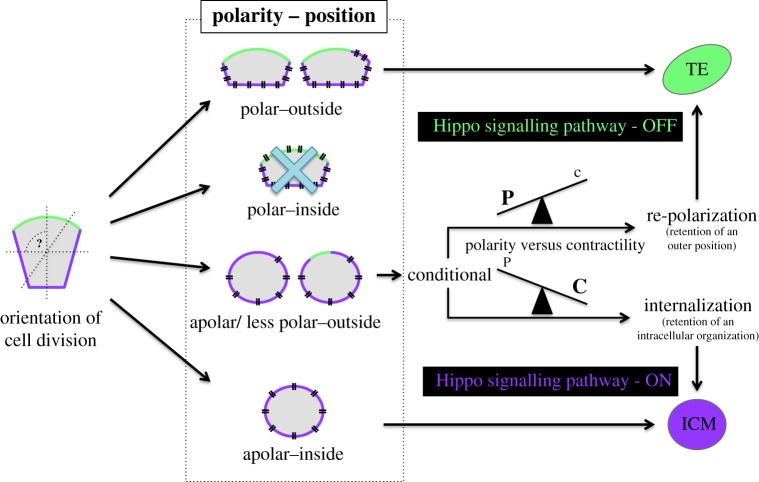
An encompassing ‘polarity-dependent cell-positioning’ model of the first cell-fate decision in preimplantation mouse embryo development. A schematic representation of the ‘polarity-dependent cell-positioning’ model showing that, based upon the orientation of a cell division (either at the 8-cell stage or in outer blastomeres at the 16-cell stage) with respect to the apical–basolateral polarity axis, daughter cells are generated with differing extents of apical–basolateral polarity and can acquire different initial positions (i.e. are subjected to different degrees of cell-to-cell contact) in the embryo. Simultaneous inheritance of a functional apical domain and an outer position upon cell division allows a cell to maintain its position, prevent Hippo-signalling pathway activation and subsequently acquire trophectoderm (TE) fate; an alternative scenario in which a daughter cell inherits an extensive amount of the apical domain and an inner position upon cell division is not observable during preimplantation mouse embryo development (see schematic ablated by a cross). In contrast, an apolar cell that initially inherits the inner position within the embryo (completely surrounded by other cells) is prevented from repolarizing and due to resultant Hippo-signalling pathway activation acquires the ICM fate. Finally, the fate of a daughter cell that inherits a small portion of the apical domain, or no apical domain whatsoever, and initially resides on the outside of the embryo, is conditional upon the balance between polarity (that acts to prevent cell internalization) and actomyosin contractility (that drives cell internalization). The absence of cell-to-cell contact at the contactless domain provides an opportunity for the cell to repolarize/enhance polarity, in order to overcome the internalizing forces of actomyosin contractility. Thus, in cases where polarity prevails over contractility (P > C), the cell retains the outside position and suppressed Hippo-signalling pathway, and contributes to the TE. However, if the forces of actomyosin contractility prevail over the inhibitory influence of polarity (P < C), a cell becomes internalized and the opportunity to repolarize is lost. Consequently, the Hippo-signalling pathway becomes active and the cell acquires an ICM fate. Note that solid green and purple lines, respectively, mark the apical and basolateral membrane domains, while dashed black lines mark the potential cleavage planes. In addition, the sites of cell-to-cell contact (indicative of the cell position in the embryo) are marked with two parallel black lines.

The main question left unanswered by either model is: what determines the fate of an outer-residing apolar/less polar cell? As it may either stay outside and repolarize or remain apolar and internalize, its fate will be entirely dependent upon which of these two processes actually occurs. The outcome most probably depends on the balance between the extent of apical polarity and the strength of the apical constriction/actomyosin contractility (acting as a mechanism of cell positioning) in any given outer blastomere. Accordingly, if the extent of apical polarity is sufficient to prevail over the effect of any inherent apical constriction, the concerned blastomere will retain its outer position and, thus, become the trophectoderm. If the opposite occurs, it will ultimately become internalized and contribute to the ICM. Indeed, whether such an inter-relational balance truly exists, and if it is possible to functionally uncouple these two components (for example, driving a polarized cell to internalize) can form the basis of future research into this most fundamental and dynamic of developmental windows, thus potentially opening the way towards a more encompassing and combined ‘polarity-dependent cell-positioning’ model (figure 4) that would be able to adequately explain how all cells within a developing preimplantation stage mouse embryo ultimately find their appropriate fate in either the trophectoderm or ICM of early-stage blastocysts. Accordingly, such a combined polarity-dependent cell-positioning model, as we propose here, stipulates that initial differences among blastomeres, in both their intracellular organization and cell position, originate directly as the result of the division orientation of ancestral/parental blastomeres, with respect to the axis of cell polarity, i.e. the radial axis of the embryo. Consequently, three conceptually different cell populations are created differing in their intracellular organization and position: polarized outer cells, apolar inner cells and apolar/less polar outer cells. The first two of these cell populations already possess an intracellular organization that is compatible with their respective relative positions in the embryo and subsequently acquire an appropriate cell fate; i.e. the concomitant inheritance of a functional apical domain and an outer position permits Hippo-signalling pathway inactivation and the acquisition of trophectoderm cell fate, while the inheritance of an inner position by apolar cells prevents apical–basolateral repolarization and allows the required Hippo-signalling pathway activation that will ultimately result in adoption of an ICM cell fate. The existence of the apolar/less polar outer cells is, however, only temporarily tolerated, as these cells will be forced to adjust either their position or polarization status, in order to acquire an appropriate fate. Thus, the acquisition of fate is conditional and the outcome depends on the balance between polarity and contractility; i.e. such cells can repolarize to remain as an endowed outer trophectoderm progenitor or internalize to become a founder ICM cell. Interestingly, the balance between cell polarity and contractility does not necessarily have to be a mere reflection of the extent of intracellular polarity inherited by a cell upon an asymmetric cell division, important though it clearly is. On this level, the polarity-dependent cell-positioning model takes into account that contractility itself may be additionally and independently regulated; for example one can imagine the hypothetical scenario in which two blastomeres exhibiting the same extent of apical cell polarity (potentially low) are distinguished by differing extents of actomyosin contractility, which could conceivably result in each cell ultimately adopting a different fate. In addition, the fact that apolar outer-residing blastomeres possess the ability to repolarize [56,81] indicates that the extent of cell polarity directly inherited as a result of cell division does not necessarily have to remain constant throughout the ensuing cell cycle. Therefore, the presence of a contactless domain provides such cells with the opportunity to repolarize, or at least increase the extent of their apical polarity from a comparatively low level. Moreover, it is the degree to which this process occurs that will dictate whether the blastomere retains an outer position or alternatively is internally repositioned. In either case, the ultimate position of a cell will be adjusted in accordance with its intracellular organization/polarization status, so that by the time the two cell lineages become irreversibly segregated (at the mid-blastocyst stage), the outer-residing polar cells with an inactive Hippo-signalling pathway will exclusively contribute to the trophectoderm, while the inner apolar cells with an activated Hippo-signalling pathway will form the ICM cell lineages.

## Data accessibility

This article has no additional data.
